# Non‐Directiveness and Authenticity in the Predictive Genetic Clinic

**DOI:** 10.1111/1467-9566.70149

**Published:** 2026-01-29

**Authors:** Shane Doheny, Rebecca Dimond, Lisa Ballard, Anneke Lucassen, Angus Clarke

**Affiliations:** ^1^ Division of Cancer & Genetics Cardiff University Institute of Medical Genetics School of Medicine Heath Park Cardiff Wales UK; ^2^ School of Social Sciences Cardiff University Cardiff Wales UK; ^3^ Clinical Ethics Law and Society University of Southampton Southampton UK; ^4^ Nuffield Department of Medicine Centre for Personalised Medicine University of Oxford Oxford UK

**Keywords:** authenticity, habermas, non‐directive counselling, predictive genetic test

## Abstract

The predictive genetic clinic is a space where counsellors use non‐directive counselling to facilitate asymptomatic patients at risk of carrying a dominantly inherited disease access a predictive genetic test. The social science literature has a history of examining practices within this clinic, but with little attention from the sociology of identity. In this paper, we highlight the importance of identity within these clinics by examining how currently healthy patients anticipate the prospect of a future identity of illness and death. We do this by examining how patients authenticate a decision to take a predictive genetic test for Huntington's Disease (HD). In deciding to take this test, a patient simultaneously asserts that they want the test, and they will be able to cope with a positive (bad news) test result. Positioning this as a claim to authenticity using Habermas, we explore authentic decision making through four themes—vouching, calibrating, reassuring and projecting. Non‐directive counselling provides space for patients to articulate the authenticity of their decision while enabling counsellors probe their decision. However, counselling risks hindering authentic decision making and may devalue the social and familial as bases for efforts to authenticate.

## 1 Introduction

In this paper we explore how healthy patients authenticate they want foreknowledge they will become ill and die from an incurable disease and how some efforts can be overlooked in the clinic. Issues about how we authenticate an identity have a long history in the sociology of medicine. Studies of identity and authentication have addressed how people identify in terms of their health, their attitude towards health and judge the unhealthy (Pelters 2024). Such studies examine changes and disruptions to the identities of people who have become sick (Charmaz 1995; Fang et al. 2024) or maintain a sense of identity despite illness (Hinojosa et al. 2008). What is lacking is an examination of identity among people who are healthy and opt to gather information on future disease and death.

The sociology of genetic counselling can add insight on the effort to authenticate an identity as one ready for presymptomatic health information. A currently healthy person deciding to take a test that may confirm presence of an incurable disease (such as Huntington's Disease [HD]) involves developing certainty the patient wants this information and will be able to cope with having it (Clarke 2019). Unlike many predictive genetic tests the result for HD has little impact on clinical management but may help patients in their own lives (Tibben 2007).

Using a critical theoretic approach, we situate authenticity as socially constructed in these clinics. Commensurate with this approach we take an abductive approach to inquiry insofar as we focus on authenticity to explain decision making in these clinics. In the following first we (2) outline HD and (3) non‐directive counselling. We then (4) outline our critical theoretic approach before (5) unpacking authenticity. Against this background, we (6) describe the methods used to gather data (7) present our analysis and (8) conclude with some reflections on efforts to authenticate.

## 2 Huntington's Disease

The clinics observed for this project were for patients at risk of HD, who can consider and discuss taking a *predictive* or presymptomatic test. HD is a progressive neurodegenerative disorder that cannot be cured that affects movement, cognition, affect and behaviour. In terms of its genetics, HD is autosomal dominant (Clarke 2019, 255), which means the gene variant resides on an autosome (not a sex chromosomes); one copy of the gene needs to contain a variant to be at risk and there is a 50% (1 in 2) chance of passing the variant to a child. For patients, knowing whether they will develop the condition has profound and long‐term consequences (Winnberg et al. 2018).

The willingness to consider predictive testing for HD relates to a sense of biographical risk connected with proximity to the disease (Cox and McKellin 1999). Many at risk of HD decide not to take the test because of worries about the impact on children, fear of an unfavourable result and a decision not to know (Ibisler et al. 2017). Those who opt for testing can make this decision spontaneously or following deliberation (Taylor 2005). Motivations for testing include a desire to eliminate uncertainty and to be able to plan one's private life (Ibisler et al. 2017). Social context also plays a role in motivating decisions to test. Patients can seek testing in response to questioning by family or friends and/or practical concerns such as personal or marital goals (Tillerås et al. 2020, 10).

In an analysis of predictive genetic clinics, Sarangi et al. (2004) distinguish three patterns in the rationale patients used to justify their decision—‘Gaining knowledge as a Basis for Future Action’, ‘Needing to know as a subjective necessity’ and ‘Downplaying What Can Be Known’. Importantly for us, Sarangi et al. (2004), (2005) view these encounters as a kind of negotiation. During this negotiation, counsellors seek ‘to ensure that clients have made decisions with sufficient consideration of all possible consequences and free from external pressures’ whereas patients respond by demonstrating an awareness of the nature of good reasons and ‘are thus likely to engineer their responses in ways that match these criteria’ (2005: 40).

## 3 Non‐Directive Counselling

Non‐directive counselling played a central role in shaping genetic counselling (Clarke 2017). For Rogers (1942), directive counselling involves counsellors defining problems, identifying their causes, and offering ideas to clarify and overcome these problems. By contrast, in non‐directive counselling, the patient defines the problem, identifies life goals and works with the counsellor on ways to achieve these goals (see Wolff and Jung 1995, 11, 12). Non‐directiveness can appear similar to shared decision making (Elwyn 2021). Both focus on deliberating decisions with patients. Shared decision‐making addresses questions of patient goals in areas where there is equipoise between decision alternatives. In predictive genetic clinics the decision outcome will have profound impact on the patient and little impact on the counsellor, and where the impacts of a yes/no decision are very different, then non‐directiveness is more useful than shared decision making (see Clarke 2017).

The concept of autonomy has been central to debates on non‐directiveness. Both value neutral and ethical views of non‐directiveness are based on a traditional concept of autonomy (Clarke and Wallgren‐Pettersson 2019; Warton et al. 2023). However, as Warton et al. (2023) note in the context of non‐directive prenatal counselling, autonomy as freedom from interference neglects the social context of decision making (see also, Horton and Lucassen 2019). But although decision making in relation to predictive genetic test for HD can be individually focused, those affected live in social environments. To protect autonomy, Oduncu (2002) suggests counsellors support patients to make their own autonomous decisions not only as a freedom from coercion, but also a freedom to make one's own decision, a freedom that relies on the beneficence of the counsellor.

## 4 A Critical Theoretic Approach to Authenticity

While mapping the complex treatment of authenticity in sociology, Vannini and Franzese (2008) nevertheless summarise authenticity as ‘about being true to one's self’ (2008: 1633) and offer advice on how research can illuminate this experience. On this view there is a ‘true’ self that may be articulated or revealed to the self that can stand at odds with society, and forms a basis for evaluating the self and motivating action. But this approach may under appreciate the role of society in shaping authenticity. Hence, sociology also positions authenticity in a social environment and examines how people use strategies to authenticate the validity of their identity (Brekhus 2020).

Seeking a critical theoretical approach to authenticity, Varga (2012) distinguishes three views of authenticity. The ‘inner sense’ view holds that we have an ‘inner being’ that we can uncover through introspection (2012: 62). For Varga (2012) the productionist view ‘emphasises [authenticity as] active involvement in constituting who we are through wishes, desires, and motivations’ (2012: 70). Both of these, Varga (2012) argues, neglect the normative dimension. To correct for this, Varga (2012: 89, 81) proposes using ‘wholeheartedness’ to conceptualise authenticity. A wholehearted commitment to a project implies the project is ‘so central to one's self‐understanding that betraying it would also mean betraying oneself’ and involves a continuity of commitment so that ‘besides wanting to reach the specific goal, we simultaneously want to continue wanting it’ (2012: 81).

Varga (2012) uses these constructions to analyse discourses of authenticity. Ultimately, this represents a transcendentalist approach to critique (see Strydom 2011, 168) insofar as this approach uses idealisations to illuminate discourse. By contrast, Habermas's constructivism locates critique inside society. Explaining this constructivism as immanent transcendence, Strydom (2011: 97, 98) distinguishes everyday (or immanent) presuppositions and transcendent presuppositions. This view assumes there are underlying presuppositions embedded within lifeworld experience that everyone can share and that are given shape and direction by cultures and ideas that transcend context. Immanent transcendent critique focuses on uncovering transcendent structures from within immanent practices (see Doheny and Jones 2021). In this paper, the relevant immanent lifeworld experience is the struggle to have one's identity recognised and is connected with authenticity as a transcendent idea focussing on the processes by which individual difference gains recognition.

## 5 Habermas and Authenticity

In a study of individuation, Habermas (1995) explores how humans stabilise individuated subjectivities in individualised societies. Through this Habermas develops a view of authenticity as, on the one hand, a performance that can be observed (1995: 168, 169) and on the other, an experience that can be understood (1995: 183–192).

### 5.1 Vouching

On Habermas's analysis, a person's claim to individuality can be observed in their performance of individuality. In performance, the person presents themselves as authentic in a social milieu where claims can be assessed (Habermas 1995, 168). We can observe a person presenting a guarantee of authenticity before others who weigh this guarantee as a ‘vouch’. As an authentic person, ‘the individual projects himself [or herself] as someone who *vouches* [italics in original] for the more or less clearly established continuity of a more or less consciously appropriated life history’ (1995: 168). This view includes the centrality and continuity elements Varga (2012) finds in wholeheartedness. However, Habermas's (1995: 169) separation of the guarantee and the vouch locates claims in social contexts.

Positioning authenticity in a social context creates the space to consider how social processes shape authenticity. But although we can observe a claim an authenticity and its reception, this does not explain the formation of the claim (1995: 170). Continuing his study of the production of an individuated self, Habermas follows Mead (1967) in situating authentic subjectivity as socially produced and socially constructed.

### 5.2 Calibrating Authentic Claims

Following Mead's analysis, Habermas conceptualises how self‐consciousness is ‘communicatively generated’ (1995: 177). This consciousness is structured using what Strydom (2015: 275) summarises as ‘an ensemble of conditions that both enable and limit’ or ‘pre‐suppositions’. For Habermas (1995: 175, 176), Mead explains self‐consciousness by separating a spontaneous subjectivity from a socially conditioned subjectivity (1995: 177). It follows then that the authentic person (what Habermas calls the ‘practical relation to self’) internalises the ensemble of social conditions as ‘an agency of self control. [With] … the … tasks of mobilising motives for action and internally controlling one's own modes of behaviour’ (Habermas 1995, 179). We summarise this form of authenticity as calibration. The individual calibrates their claims to authenticity to encompass and control their motivations mindful of their enabling and limiting social conditions.

### 5.3 Reassuring of Authenticity

Following Habermas's analysis, a second level emerges as individuals internalise conventions. Habermas explores Mead's distinction between the ‘I’ and the ‘me’ to demonstrate how the internalisation of language and culture affects authenticity. The ‘I’ refers to a spontaneous, creative, impulsive, innovative and pre‐social force whereas the ‘me’ refers to the internalisation of language and culture. Internalising norms, rules, cultures and symbols in the form of a ‘me’ leads to two conclusions. One is the authentic person's capacity to follow or break rules flows from the incorporation of norms into the personality system in the ‘me’. The second is the social conditions exert power over the individual. Internalising social conditions in a ‘me’, the individual internalises the norms of ‘a *particular* collective will’ (1995: 182, italics in original) which limits the capacity of the ‘I’ to recognise its own motivations and priorities. The result is a kind of ‘blind subjugation’ so that internal controls chastise norm breaking and compel norm compliance with little justification.

The issue here concerns the effect internalised social conditions have on the capacity to reveal and act on the authentic self. The authentic person has the capacity to decide for themselves whether or not to follow rules, but to have this capacity implies incorporating social conditions in a way that supports people to decide for themselves. However, internalisation involves a degree of acceptance. For these reasons we characterise this form of authenticity in terms of reassurance. The individual wants reassurance they are authentic, but this reassurance is challenged by internalised social conditions.

### 5.4 Projecting an Authentic Self

The context shifts with the shift to the language and culture of complex post‐traditional societies where the individuals are expected to decide for themselves about the norms and conventions they want to live by. Societal differentiation, for Habermas, ‘burdens’ the individual with the task of defining their own ‘life project’ while increasing differentiation of life projects undermines the possibility of stabilising individual identity using the cultures or norms of particular communities. Instead, personal will finds stability by engaging with anticipated communities (1995: 184). This post‐conventional identity builds on the conventional identity where the individual becomes conscious of authentic individuality by appropriating the language and culture of their community. But in the post‐conventional context, the claim to authenticity is structured by projections. Instead of identifying with the cultures and expectations of a real community that here are experienced as limiting, this post‐conventional identity positions itself in relation to all communities, and projects a communicative context. For Habermas, this shift has a social and a cultural effect on authenticity. In terms of its social implication, the post conventional claim to authenticity is constructed as unique and irreplaceable. This means positioning oneself in relation to a projected form of society that would understand the nature of the claim. In terms of its cultural implications, the social conventions of specific communities no longer have power over the individual. Instead, this individual ‘*projects* the context of interaction’ (1995: 187, italics in original). By projecting the communities the self anticipates interacting with, an authentic identity develops reflective awareness of its claims to authenticity and of the properties of the community that would recognise these.

## 6 Methods

The data presented here was collected as part of a project on how patients decide to take tests of limited clinical utility. Our data included observations of 15 cases involving patients at risk of HD. These cases include observations made of pre‐clinic, pre‐test, test and results clinics. We did not include any post‐result clinics.

Potential participants were identified by clinicians and sent an information sheet and an invitation to participate. At the clinic, the clinician secured oral consent from patients and the first clinic visit was audio‐recorded; consent was formally documented at the end of this appointment. This meant that counsellors approached patients for inclusion in the study. However, because they sent letters and information sheets to eligible patients before the clinic, then raised the possibility of participating at the beginning of their first clinic with the patient, counsellors were not pre‐selecting patients based on previous knowledge. Approval for the project was obtained from an NHS Research Ethics Committee (REC name) and the Research & Development Office of each NHS trust involved. Non directive counselling forms a central part of genetic and now genomic counsellor training (see Clarke 2019, 18–20). The participating genetics specialists were practitioners with an interest in the social dimensions of genetics. As such, they were reflective about their counselling practice and tolerant of sociological observation but, equally, may provide a particular view of counselling practice. Nevertheless, as the purpose of this study is to identify the factors enabling patients make their own decisions before new genomic technologies are introduced that may complicate decision making, the cooperation of clinicians and counsellors attuned to such interests is of vital importance (see Ballard et al. 2025). The analysis focuses on ethnographic observations alone. Patients also participated in interviews and some completed a diary that have been reported elsewhere (Dimond et al. 2022; Ballard et al. 2025).

The analysis below is based on the predictive genetic clinic consultations with 15 patients at risk of Huntington's Disease. Observations included 21 clinic consultations. These included four pre‐clinic meetings, 11 pre‐test clinic sessions, five test clinics and one results clinic. These consultations took place between 2018 and 2020 in South and South‐West England and in Wales. Seven of the patients were female, eight were male. Ages ranged from 16 to 69. All but one of these patients proceeded to take the predictive genetic test. This makes this cohort unusual insofar as patients are often ambivalent and procrastinate their test decision. Three patients were known to counsellors before they were invited and so most were not pre‐selected. Hence, we cannot explain the tendency of this group to proceed to the test. This sample was dealt with as normal within clinics and themes of ambivalence were also prominent (see Ballard et al. 2025).

Observations included consultations led by three other clinical geneticists (labelled CG1 through CG3) and of three genetic counsellors (labelled GC1, GC2 and GC3, all patient names are pseudonyms). 10 of the 20 clinics were led by one clinical geneticist (CG2) because this clinic made up a large part of this geneticist's workload. The study included pre‐clinic sessions in which counsellors gather information about the patient, pre‐test clinics, test (or blood draw) clinics and one results clinic. This structuring of clinics follows policy guidance on counselling HD patients (MacLeod et al. 2013). The analysis here focuses on the eleven pre‐test clinic sessions.

Our analysis follows the abductive mode of qualitative reasoning. Abductive analysis starts with the perception of similarity in observed phenomenon, but where the structure of the similarity is neither anticipated (as in deduction) nor observed (induction) but ‘guessed at’ to explain observations that are otherwise difficult to explain (Tavory and Timmermans 2014, 37). In our data, authenticity was discussed once and was not coded in coding rounds. Through rereading the data, the lead author formed the abductive view that discussion of the decision to take the predictive test was shaped by a claim to authenticity (understood as a claim to initiate a test the consequences of which the patient will be accountable for), and work commenced to explore the authenticity in this dataset (see Tavory and Timmermans 2014; Strydom 2011, 155).

## 7 Analysis: Negotiating Authenticity

Many patients articulated a desire to know their inheritance. In these cases, counsellors used non‐directive counselling to challenge patients. Patients responded by providing forms of guarantee of their willingness to live with their decision and any consequences. In this sense, the patients drew on the rationality of authenticity.

### 7.1 The Use of the Vouch in the Clinic

Relations of power permeate these clinics. Patients are unable to access this test without the support of a clinical geneticist, and are expected to respond to questioning in the clinic. The clinics themselves are organised to incorporate an interruption separating a pre‐test clinic and a test clinic. The interruption is supposed to ensure the test clinic is arranged by the patient, but was also by used by clinicians to set agendas for clinic appointments. The following segments are taken from the closing moments of Susan's pre‐test and the beginning of her test clinic. In the pre‐test clinic, the counsellor explains the transition to the test clinic as involving a ‘checking’ of the patient's thinking.

G2 So nothing else specific to think about today.

[five turns omitted]

G2 So I think next time will be much simpler. Well probably, by which I mean that we'll be checking that you're in the same place, and wanting to go ahead. And we can think about any questions that crop up, but I think we've done most of the talking really. And as long as you're clear that you feel the same, and then we'll examine you and go through the consent and the blood taking. (Susan, pre‐test clinic)

This notion of ‘checking’ is taken up again in the test clinic:

G2 Sorry, so coming back to … so yeah, last time we had a pretty full discussion, I think today was a chance to check that you're still of the same mind about things.

Susan Mm, mm.

G2 Yeah, you are, yeah?

Susan Yeah, and I noticed in the last letter that came out in the like… there was a comment that um, you know, I should potentially consider changing… pushing things back, because of Mum passing last year.

G2 It was… it was something we hadn't talked about, and I just thought, when I was doing the letter, would that be something you'd want to… to think about, or not? (Susan, test clinic)

The opening question in both segments uses non‐directiveness to invite the patient to articulate her thoughts. Central is the idea of the ‘check’. The object of this ‘check’ was the patient's claim that the decision was hers and remained the same over time. Although Susan guarantees a commitment to a decision in one clinic, by treating this guarantee as a provisional assertion, a vouch, the counsellor uses agenda setting power to create space for revision and to establish counselling as the purpose of the clinic. In her response, Susan challenges reasons that undermine her guarantees offers.

Choosing Susan's emotional stability following the death of her mother addresses individualises how she may respond to a positive result in isolation from socio‐economic, community or familial considerations. Arranging clinics around the vouch meant bracketing the social and economic and prioritising the personal and emotional. This created problems for some patients.

GC3 [omitting turns on timings of clinics] I just actually, when I looked at your referral, it says you're, it's quite difficult for you to come to [Coastal city], is that right?

John It is yeah.

GC3 Yeah.

John That's what I was gonna talk to you about, is the problem for me, is it's gonna sound really weird, but I'll try and keep it simple, but um, obviously every single bit of penny is counted for in my house, so by this appointment today, I've lost 60 pounds just in 1 week.

[nine turns discussing financial implications of clinic attendance omitted]

John So obviously what I'd love to happen, is part of me come here on the quiet, to be honest. Um my mum's passed me down underactive thyroid, gone, me mum's passed me down [unclear], just had surgery done, that's gone. The chances of getting Huntington's knowing my luck, probably got it. That's just the obvious. I've probably got it. So you know.

GC3 Um it's interesting you think that isn't it.

John So you know.

GC3 Cos I literally can't give you. […] (John, pre‐clinic)

This is a pre‐clinic where counselling focuses on gathering information relevant for the pre‐test clinic. In this segment the counsellor raises the topic of issues John faces in attending the clinic. John underlines the costs of attending. As the segment continues John outlines reasons that lead him to conclude he carries the gene change suggesting he expects he will receive a positive result and counselling will not benefit him. The counsellor emphasises the role of counselling (‘it's interesting you think that isn't it’) focussing on John in isolation from his familial and economic situation.

Bringing a patient through a series of clinics where counsellors treat efforts to authenticate as a vouch focuses on the individual in isolation from their familial and economic situation. This treatment of a patient's guarantee as an effort to vouch in the context of a sequence of clinics in which clinicians have power over timing and purpose and require that patients engage with counselling, means the vouch becomes a key to the use of power in this clinic.

### 7.2 Calibrating a Claim to Authenticity

The claim to authenticity was not always successful. In the clinics, we observed one case where a patient struggled to be persuasive. In the following, we follow the emergence of this patient's claim over three segments.

#### 7.2.1 Rebecca

This case involves a young woman, Rebecca, who came to a clinic with two young children and her mother.

G2 'Cause some people are very clear straight away…

Rebecca Yeah.

G2 … and other people need a bit more time to think about it.

Rebecca I've had 6 months, I've thought about it 6 months ago, so… I'm pretty sure that I want to go ahead.

G2 Yeah, okay, I mean you, you, I think your GP [clicks tongue] sent you to us first like a couple of years ago.

Rebecca Yeah, yeah, and then, I don't know, I think back then I, I don't think I was ready…

G2 No.

Rebecca … and, and now I think, after I've had my little 'un, erm, I'm ready to go ahead now.

[four turns omitted]

G2 Yeah…[pause]…, yeah and what, what's made you change your mind do you think?

Rebecca Just the way that I've seen my dad recently, over the past 6 months he's gone downhill really, and it's just something that's not, it's playing on my mind, I just, I would rather find out now rather than later.

G2 Mm.

Rebecca Okay. I'm pretty quite headstrong so… (Rebecca, pre test clinic)

The counsellor initially sets a scene that avoids a predetermined response. Rebecca uses uncertain language (‘pretty sure’) and a past present formulation (‘back then’ and ‘I'm ready to go ahead now’) to indicate readiness. The counsellor asks about a previous appointment that Rebecca did not attend using an open question which allows Rebecca to select a response. Rebecca gives a reason (‘I've had my little 'un’ (her baby)), but does not elaborate, instead talks of wanting the result to stop worrying about the condition. She refers to her father's deterioration as context and suggests the idea she might have HD is influencing her internal state (‘it's playing on my mind’) shaping her preferences (‘I would rather find out now rather than later’). The combination of non‐directive and open questioning therefore creates space for Rebecca to reveal an inner turmoil. Using Habermas, we can see that Rebecca is working to formulate a guarantee. She provides a claim for authentic decision making by pointing to time she has spent thinking, her observations of her father and her application of these to herself. But her reference to inner turmoil adds a complication.

Following a discussion of care for Rebecca's father, the following exchange takes place. Here, the counsellor focuses on how HD is ‘playing’ on Rebecca's mind. Again, the counsellor uses agenda setting power to raise a topic.

G2 Yeah, yeah. So [Patient's name], when someone says like, to you, that it’s in your mind an awful lot? So that would be like every day?

Rebecca Yeah, I do think about it quite a lot and especially after I had my, my youngest one and I, now that she's turned a year old, I just think, no, I want to do it for them as well. It has been on my mind for the past 6 months.

G2 Yeah, and when it comes into your, in your mind, what are you thinking?

Rebecca It scares me.

G2 That you might be like that [referring to the patient's father]?

Rebecca Yeah.

G2 Yeah, and, oh yeah, I, I suppose what that makes me think, you know, is that, that's what you're worried about now, when this is something that might be bad for you. […] (Rebecca, pre test clinic)

Here, the counsellor's questioning remains non‐directive. He focuses on Rebecca's internal life but in a hypothetical way, asking about thoughts that accompany reflection on the test. The response does not describe a thought, but Rebecca's emotional state when thinking about HD. The counsellor requests confirmation this fear is driven by the prospect of becoming like her father and voices concern about taking the test at this time. Using the Habermas framework, we can see both that the counsellor uses non‐directive counselling to explore how Rebecca vouches for her decision and that Rebecca's internalisation of her social conditions is creating a sense of fear within her. In the following, Rebecca undermines the continuity of her commitment to wanting this information.

G2 It's about you, for a while. It will make a difference once you maybe would have to say more to, to, er, the children but for a few years it probably won't make that much difference to anything practically.

Rebecca I think if I don't do it now then I probably wouldn't do it in a few years 'cause I think like…

G2 Why not?

Rebecca … 'cause I've dealt with everything before now, so I just think I just want to get it over and done with [laughing].

G2 Oh no, I get the message that you want, you want to get it over and done with.

Rebecca Mm.

G2 But that's a funny way to put it.

[Omitting four turns on reservations about the test]

Rebecca Yeah, I, I, obviously it's not a nice thing to go through, I know that but I just think if I don't get it done now, I, I probably, I don't know. I don't know, there's a lot of things that goes on, that's really hard to explain [laughs]. All I know is that I've put it, like I say I've just, I've put it off once, I wouldn't want to put it off again.

[four turns omitted]

G2 And you're telling me that if you, it sounds like you've screwed yourself up to do it now. (Rebecca, pre test clinic)

Here, the counsellor points to the limited difference having this information will have for Rebecca. Rebecca responds that if she does not do the test now, she may not want to do it in another few years. A key utterance is ‘I've dealt with everything before now, so I just think I just want to get it over and done with’. She does not explain what is meant by ‘everything’, but earlier she points to concerns about the health of members of her family, so the combination of ‘dealt with’ and ‘everything’ may suggest these concerns have been managed, and she has an opportunity to focus on herself. This opportunity to focus on her own health is also placed inside a temporal frame. To want to get learning of her genetic risk ‘over and done with’ suggests a desire to put this discovery into her past. This undermines the expectation that her future self will still want to have had the test, if she receives a bad news result. In effect, she has not calibrated the effect her decision is likely to have.

Weighing a guarantee as a vouch involves a use of power. For the counsellors, an acceptable guarantee focused on the individual's capacity to guarantee that they will continue to want the test result, even if it is bad news. But neither the Habermas framework nor the counselling considers how a guarantee may be based on group structures. Relevant is how Rebecca indicates that the space she has to consider her health will change. At a number of points, Rebecca indicates changes in her family that prompt her to want this test. Having had her second child, wanting the test for her children and, in the third segment, she suggests complex situations without elaborating. It is at least plausible that Rebecca acted like a linchpin for a family with multiple health problems so the basis of her claim to authenticity may have been that her family needs to know her predictive status to prepare for a future without her.

The problem is that Rebecca's claim to authenticity is inconsistent and not well calibrated. However, the recommendation that Rebecca return for further pre‐test counselling focuses on Rebecca's personal inconsistency potentially overlooking a familial basis of her authenticity claim. Insofar as Rebecca offers a familial sense of authenticity her guarantee may incorporate a need to plan for the future of a family experiencing severe health related stress. A more adequately calibrated claim to authenticity may draw together her fears for herself and the interests of her family.

#### 7.2.2 Kate

The following involves a patient who asserts she wants to know without indicating how she will use this knowledge. At the time of her clinic, Kate was a minor. Although Kate can request the test, available guidance recommends counsellors take an individualised approach while exercising caution in offering her this test (MacLeod et al. 2013, 223).

GC3 […] At the moment are you thinking about testing, or are you just wanting information?

[Omitting two turns]

Kate I dunno, I just always have, […] um and then when I, she [patient's sister] was like, ‘do you wanna get tested?’ And I was like ‘yeah, definitely’, she was like ‘why though?’ I was like, ‘cos I just wanna’. I'm the person that likes to know things like even, just anything (chuckling), um and um, she's like, if she doesn't agree with what I say, she definitely tells me that she doesn't agree (chuckling).

GC3 Okay.

Kate So she's like, ‘I wouldn't do it if I was you’. I was like ‘okay but it's not your choice’, so she don't want to, but that's not a problem (chuckling).

[Omitting eight turns]

GC3 […] is it gonna change, what, what do you think it would change for you knowing?

Kate Well I've always said that if the test came back positive, then I think that's even more reason to live my life while I still can, before like anything, the symptoms come.

GC3 Okay.

Kate Because what, what's the point in just sitting, doing nothing.

GC3 And just to pay sort of devil's advocate, is that not something you could do anyway, live life to the full?

Kate It is, it is, but I dunno, I just wanna know, I don't really have like, you know, like a reason. I just wanna know. (Kate, pre‐clinic)

The counsellor opens with a non‐directive enquiry about Kate's objectives. Kate describes her motivation by recounting a conversation with her sister. Earlier, Kate reported her sister had begun predictive genetic counselling and decided against testing. In the reported conversation, Kate's sister asks about Kate's motivation. Kate responds that she “just wants to know”. Challenging this, the counsellor asks for information about what knowing will change. Kate responds with a claim to authenticity indicating this knowledge will motive her to be more active which the counsellor again challenges. Here, the sense of calibration becomes clear. Kate has conveyed a claim that knowing she is presymptomatic for a life limiting illness, she will live her remaining life to the full, has met resistance. This claim may seem convincing from someone who has experience of themselves and their responses in different scenarios and can see how to control their likely response to a positive result, but may seem less convincing from a minor. Kate ends by expressing uncertainty, undermining her claim for authenticity. A more adequately calibrated claim for Kate might take into account the choices she faces at this stage in her life, and how a result would help her to make other decisions. A counselling mindful of calibrating might both challenge the limitations of Kate's claims, and offer advice on the structure of a more convincing claim.

In these cases, non‐directive counselling created space for patients to present a language of authenticity. Although there can be little doubt that these patients wanted to have the test, both struggled to calibrate their claim to authenticity. What our analysis also shows is how counselling may not fully appreciate how these patients calibrate their claim. The social and familial part of Rebecca's claim is not featured and Kate is unable to clarify the dimensions of her claim. Non‐directive counselling may want to go further than to challenge these patients by helping them clarify the aspects their claims.

### 7.3 Reassuring of Authenticity

On Habermas' narrative, the sense of authenticity is both enabled and challenged by one's social environment. In an environment characterised by convention, the individual wants reassurance that they really are making a decision that is authentically their own.

#### 7.3.1 Harry

Harry's pre‐clinic exemplifies how counsellors sometimes requested clarification that the decision was the patient's own. The following exchange takes place following discussions of conversations Harry has had with his friends. Here the counsellor questions how Harry internalises these conversations.

CG3 Now the reason I'm asking is cos you do tend to find that people get very mixed views from people and sometimes that can be actually quite hard in, in making your own mind up, because you're getting so much input.

Harry Yeah, yeah, I know some people can, you know, quite strongly wouldn't want to know for example, something like that. […] But no, yeah everyone around me seems to be on a similar wave length.

CG3 Yeah, yeah and does that make you feel that it is your decision you're making though or is it again, it can be quite a difficult thing to?

Harry Yeah, I, I don't feel as though I'm pressurised into it, I don't feel as though I have to you know, I, I have to do one thing to appease … (Harry, pre‐clinic)

Harry had discussed HD and the test with his friendship group. Here, the counsellor focuses on pressure arising from ‘social relations’ or shared ‘pre‐suppositions’ (see Strydom 2015). Harry's friends cannot force Harry to take the test, but they can create pressure through expectations or recommendations. The counsellor points to these pressures as “mixed views” and ‘getting so much input’. Harry's first deflects using general observations so the counsellor requests assurance that the decision is authentically his (‘does that make you feel that it is your decision you're making’). Harry responds to assure he is not ‘pressurised’.

#### 7.3.2 Jennifer

This clinic involved a patient who was well known to the counsellors from her time supporting her father access his predictive test. In this clinic, we focus on the impact of social conditions on Jennifer's partner, Dennis. The following extract begins with a counsellor addressing Dennis on his knowledge of HD. The counsellor situates her inquiry in relation to the idea that many people seek knowledge upon learning of their risk.

G1 Have you read much about the signs and symptoms of Huntington's Disease? Cos some people go away and read lots about things.

Dennis No, I haven't no. No, want to obviously, er, want to have the test first, before even, er, bogging ourselves down with something like that.

Jennifer I know a bit about it, obviously, from the thing from Dad, but I don't know, I just felt it was important that you knew a little bit about it cos obviously, obviously with us getting married and everything like that, obviously you're going to need to know in terms of what kind of thing, from all you said, generally people around you tend to notice it earlier than the person who's got it themselves in terms of any signs of anything then, really, because you don't recognise it in yourself as much do you then, you know?

G1 [Overlap] No.

Jennifer So… But, um … I don't know. I think, again, because we kind of focused on Dad getting tested and now coming round to us then, really. So it's only now we're starting to kind of have any significant conversations about it as such then, really. You know?

Dennis [Overlap] Right.

G1 [Overlap] yeah.

Jennifer So… cos I didn't think it was relevant up until now when… I think as Dennis said, until we have a definitive test it will be even more relevant or not relevant at that time really, but I don't know if he wanted to hear what could happen in case, or…

Dennis [Mumbles] are you… know, you said…

Jennifer I know but [overlapping speech]…

Dennis Yeah, it might be worth knowing about, yeah.

G1 So in general there's… the main feature that people kind of have always recognised is, is, is a movement disorder. […] (Jennifer, pre test clinic)

Initially Dennis defends having not observed the norm indicated by the counsellor (that upon learning they might be affected people read about the condition). His response is a statement of fact, ‘no, I haven't’ and then searches for an excuse to relieve him of responsibility by suggesting that he was not fully aware of the need to learn. Jennifer takes over and addresses Dennis (using ‘us’), acknowledging she has learnt more than he about HD, and shifting discussion from the clinic to the lifeworld context where the couple are soon to marry. In this context, the issue is living with someone who may become affected which Jennifer hedges (‘just felt it was important that you knew a little bit about it’) and deflects with an idea she attributes to the counsellors (‘from all you said’) that symptoms are first noted by those around the patient possibly to give Dennis a role, to which the counsellor provides minimal agreement. Jennifer then addresses the counsellors (using ‘we’) to explain Dennis's unawareness, highlighting how their context changed mentioning the idea that they did not need to learn about HD until they had a positive presymptomatic result. There is then a momentary conflict. Dennis points to an earlier conversation (‘you… know, you said’) possibly as justification for him delaying learning about HD. Jennifer indicates agreement and an overlap renders the exchange unclear. The conflict is resolved as Dennis indicates agreement presumably to know more about HD. The counsellor does not challenge or comment, but begins outlining the test.

The difficulty for Jennifer and Dennis is that the test is relevant for them as a couple. At the time of the test Jennifer and Dennis were cohabiting and planning to marry. Their expectations of marriage were not discussed, but later they seek counselling on family planning options. In the above, Jennifer frames the clinic as increasing Dennis's awareness of HD. Although the decision to take the test is hers, they will both be affected by the result and bringing him to the clinic ensures that he can also make an authentic commitment to this decision in the face of new pre‐suppositions about the life they might expect together.

In societies where people are expected to make decisions about themselves based on personal identity and authenticity, it can be difficult to consider how people can find their choices constrained by convention. Dennis and Harry provide examples of situations where the power of norms become topics. Counselling for Harry involved seeking assurance that the decision to test was his and that he was not subject to undue pressure. For Jennifer and Dennis, the clinic provides an opportunity to ensure Dennis has sufficient information so that can be reassured he is making an authentic decision about his life with Jennifer. These cases highlight the importance of the normative in these clinics. Harry's counsellor and Jennifer show how both counsellors and patients can skilfully use clinics to address these issues.

### 7.4 Projecting Space for an Authentic Self

The third form of authenticity identified by Habermas situates the authentic self in relation to complex societies. Here, the self sets out who they are and the kind of person they want to be, but presents this version of themselves before a projected community.

#### 7.4.1 Richard

In the following example, a patient (Richard) thinks about how a positive test result could prompt him to lead a more fulfiling life in conversation with Carys, his partner, in the clinic.

Richard Yeah. I'm hoping, it's, you know, if it's a bad result maybe it'll change my life and I'll live life to the fullest and I don't know, become a diving instructor and… [laughs]…

Carys With your ears?

Richard Yeah, with my ears, yeah.

Carys Maybe a carpenter [laughing].

Richard Yeah, yeah, all sorts of life‐changing and positive things, you know, but yeah, we can all hope.

G2 Well you can do that either way, I mean…

Richard [Laughing] yeah, I could do it either way, yeah, it's getting the motivation to do it, anyone can do it but I'm just too, I'm too afraid, I'm too afraid of change.

G2 Change.

Carys You don't like change… I wonder who you follow.

G2 Saying that is interesting in somewhat of taking this step because potentially it's a big change. […] (Richard, pre test clinic)

This segment begins with Richard discussing how he could live a fulfiling life. Richard's guarantee's is that he wants to lead a good life and a test result may propel him to overcome personal obstacles and realise this life. Clearly, this claim parallel's Kate, and the counsellor points out that Richard could achieve this without the test. Richard is in his 40s and so has more experience of himself than Kate has of herself, and he combines his claim to authentically want the test with doubts about how he will deal with a result. In this case, the counsellor responds with support. Although Richard says he fears change, the counsellor notes how gathering a result will involve some change.

Richard's claim to authenticity is questionable. Richard holds that a positive test result would motivate him to live a better life. In the following Richard suggests that finding that he is presymptomatic for HD might lead him to adopt problematic behaviours.

Richard Yes it is, I think I need some time off if I get a bad result. I end up going into work late drunk one morning and saying something…

G2 Oh you don't want to do that.

Richard … erm, tell someone how I really feel about that.

Carys I wouldn't let you to do that.

G2 No, you don't want that, but on the other hand sometimes then if you can get yourself to go back to work then it can fill in time and…

Richard Mhm.

G2 … stop you brooding too much.

Carys Yeah, a distraction…

G2 That's right, so it's, there's a balance isn't there.

Richard Yeah, I'm very good at moping, champion moper, I am. (Richard, pre test clinic)

Richard is trying to articulate a claim to authenticity to the effect that if he receives a bad news result, he will still want to have that result and such a result will propel him to living a more fulfiling life. But Richard is also conscious of limitations in his capacity to control his own behaviour. In this case, the counsellors emphasise the patient's social context. The fragility of the authenticity claim as enabling a fulfiling life is backfilled by work and family.

#### 7.4.2 Sandra

In one consultation, a patient described how she had already made changes to her life in anticipation of a positive test result.

G2 Preparing for the future longer term, do you think it would change much in the shorter term?

Sandra Uh huh, um, I mean the, you mean immediately after the result or?

G2 Potentially, in the next few years, is there, is there stuff that you would do differently if you knew for certain?

[Sandra's partner describes how the couple had already gone on ‘round the world trips’]

Sandra (Chuckling) so yeah we did that, changed my job, I thought ‘oh I don't really like this profession’, changed to be a [Professional], so I spose the kind of things.

G2 Yeah so you've done some of the stuff already?

Sandra Yeah I think that was probably a, a big life changer, I had some boring admin role, and I was like no I can do research so I retrained to be a [names profession] started a Degree, moved to an area that we wanted to live in, and my parents moved there as well, so that was about kind of setting up for the future. So I guess I've already made some structural like life changes, with that kind of thinking of, is this the life that I wanna continue to lead and yeah. (Sandra, pre test clinic)

Here, the counsellor begins with a non‐directive inquiry requesting information on possible short‐term effects of a positive test result. For Sandra, the prospect of becoming affected by HD in mid‐life prompts her to consider who she wants to be in a radical way. She wants to be a person who has seen the world and has a job she finds interesting. For Sandra the decision to have the test was based on her desire to plan her life and to make decisions about her use of remaining healthy years.

These patients consider who they want to be in the future, and the counsellors use non‐directive questions to address this projected self. For Richard a positive test result might prompt him to change his life and to become more of the person he wishes to be but could result in a loosening of the internal controls brought by convention. Sandra has already changed her life so that the actual communities she engages with better reflect the kind of person she sees herself as. Sandra has begun to live as she would like in part because of the possibility of a positive test result. Counselling consisted of allowing space in which these patients could articulate these aspirations and, for Richard, would involve shoring up this orientation.

## 8 Conclusions

This paper adds to the small but growing literature on the decision making of patients at risk of diseases for which presymptomatic testing are available (Sarangi et al. 2004, 2005; Tillerås et al. 2020) by situating the decision to take the predictive test as an authenticity claim. Taking Habermas (1995) approach, we investigate authenticity as a claim made in communication with another. From an observer point of view, a claim to authenticity guarantees enduring commitment to a plan. While listening, a second person evaluates these guarantees as efforts to ‘vouch’ or promise to stay committed to an identity. The understanding of authenticity we take from Habermas encapsulates the participant point of view with three activities. First, authenticity means calibrating claims to authentic identity in a way that articulates motivations and internal controls while conscious of prevailing social conditions. Second, under pressure from convention the individual wants to reassure themselves they are acting authentically. Third, in post‐traditional societies, people project the community or society that could comprehend their claim to authenticity. One shortcoming of this framework is that it focuses on the individual. Habermas (1996) has developed a framework that can analyse collectivistic authenticity claims in the context of the democratic constitutional state. But at the collective level, claims to authenticity raise complex questions of justification (see Cooke 1997). Future work could explore linking these frameworks and how particular claims to authenticity could gain broad based appeal.

Non‐directive counselling provides tools to encourage the patient to articulate the authentic basis of their decision and to challenge and probe a guarantee as an effort to vouch (Kessler 1997; A. Clarke 2017). Probing a commitment presented as definitive treats commitment as something like a promise. By separating pre‐test and test clinics the predictive genetic test clinic is organised in a way that enhances the vouch. Counsellors can use the vouch to check on the authenticity of a claim to wanting this test result. However, treating the definitive as a promise can be challenging for patients who find it difficult to attend a sequence of clinic in which they are expected to account for their decision to have this test.

Within the clinics, patients presented their decision to have a test as authentically their own. Counsellor challenges of these claims could sometimes focus on the patient and discount family as the source of a claim to authenticity. Second, non‐directive challenges sometimes seek to destabilise and probe what is presented as definitive claims to authenticity in decision making. While deconstructing may prompt further reflecting, counsellors could offer support by outlining a convincing claim. Third, in a society where authentic individuality is expected, our analysis highlights the skill with which both counsellors and patients address pressures to conform. Nevertheless, it remains important to check patients are authentic in their position as relevant aspects of their situation changes. Finally, authenticating unique identities involves projecting a social context to situate the identity claim. Non‐directive counselling was particularly well suited to allowing space for patients to articulate these identities, but our analysis suggests these claims can be fragile and require support. Overall, our analysis shows that counselling in these clinics does not focus on supporting patients to decide to take the predictive genetic test but on how they account for the authenticity of their decision.

## Author Contributions

**Shane Doheny:** conceptualization, methodology, investigation, project administration, formal analysis, funding acquisition, writing – original draft, writing – review and editing, data curation. **Rebecca Dimond:** writing – review and editing, funding acquisition. **Lisa Ballard:** writing – review and editing, methodology. **Anneke Lucassen:** funding acquisition, writing – review and editing. **Angus Clarke:** conceptualization, funding acquisition, writing – review and editing, project administration

## Funding

This project was funded by the Economic and Social Research Council (Reference: ES/R003092/1).

## Ethics Statement

The study was conducted in accordance with the Declaration of Helsinki, and the protocol was approved by the Wales Research Ethics Committee 1 (REC reference 18/WA/0127).

## Consent

The authors have nothing to report.

## Data Availability

Information on the data underpinning this publication, including access details, can be found in the Cardiff University Research Data Repository at https://doi.org/10.17035/cardiff.31109563.
